# Is Transcriptional Regulation of Metabolic Pathways an Optimal Strategy for Fitness?

**DOI:** 10.1371/journal.pone.0000855

**Published:** 2007-09-05

**Authors:** Carl Troein, Dag Ahrén, Morten Krogh, Carsten Peterson

**Affiliations:** 1 Computational Biology and Biological Physics, Department of Theoretical Physics, Lund University, Lund, Sweden; 2 Microbial Ecology, Department of Ecology, Lund University, Lund, Sweden; Tata Institute of Fundamental Research, India

## Abstract

**Background:**

Transcriptional regulation of the genes in metabolic pathways is a highly successful strategy, which is virtually universal in microorganisms. The *lac* operon of *E. coli* is but one example of how enzyme and transporter production can be made conditional on the presence of a nutrient to catabolize.

**Methodology:**

With a minimalist model of metabolism, cell growth and transcriptional regulation in a microorganism, we explore how the interaction between environmental conditions and gene regulation set the growth rate of cells in the phase of exponential growth. This *in silico* model, which is based on biochemical rate equations, does not describe a specific organism, but the magnitudes of its parameters are chosen to match realistic values. Optimizing the parameters of the regulatory system allows us to quantify the fitness benefit of regulation. When a second nutrient and its metabolic pathway are introduced, the system must further decide whether and how to activate both pathways.

**Conclusions:**

Even the crudest transcriptional network is shown to substantially increase the fitness of the organism, and this effect persists even when the range of nutrient levels is kept very narrow. We show that maximal growth is achieved when pathway activation is a more or less steeply graded function of the nutrient concentration. Furthermore, we predict that bistability of the system is a rare phenomenon in this context, but outline a situation where it may be selected for.

## Introduction

Transcriptional regulation of effector genes is a highly successful strategy, as evidenced by our tendency to ask how rather than whether a gene is regulated. A very natural place to study gene regulation is in the metabolism of the cell, and then specifically in the regulation of genes that code for enzymes and transporter proteins. Here, the function of regulation is quite clear: expressing the right genes at the right time will enable the cell to make the most of the resources within its reach, by maximizing the uptake and use of rate-limiting resources such as carbon and energy.

In unicellular organisms like *E. coli* and yeast, the benefits of a well-adapted regulatory system are readily quantified, as the fitness of an individual can be estimated by its growth rate in culture. A number of studies have explored how regulation of metabolic pathways affects the growth rate of microorganisms, both in the steady state and in response to changes in the local environment. In a typical experimental setup, *E. coli* is grown in a chemostat for some period of time, with one or two carbon sources present at levels that are perturbed at some point in time (see, e.g., [1]–[3]).

Over evolutionary time scales, regulation must provide a fitness benefit that offsets the costs of maintaining the regulatory system. Most immediately, precious resources will be spent on synthesizing transcription factors and replicating extra DNA, rather than going directly into growth of the cell. However, this cost can easily be dwarfed by the cost of a failure to regulate gene expression optimally, as enzymes are typically produced at far higher rates than transcription factors.

There is also an entropic cost involved in maintaining a regulatory system, stemming from random mutations that tend to destroy transcription factors and binding sites. As elucidated by Savageau [4], for functioning regulation to be present in the wild type, the population of that genotype must offset losses due to mutations by having a higher grow rate than the mutants with broken regulation. The design of the regulatory system affects the growth rate not only when the system is intact but also when it is broken, which in realistic situations can severly constrain the regulatory options. This is “survival of the flattest” [5] at work.

In the case of the *lac* operon of *E. coli*, a well-studied system for detecting and metabolizing lactose, it is known that the overall effect of expressing the *lac* genes in vain is a drop in the growth rate of as much as 5% [1], [6]. It has been argued, based on the cost in energy and carbon, that a number around 0.2% would be expected, and that the difference is more or less specific to the *lac* operon [7]. Utilization of lactose, when present, has a positive effect on the order of 10–15% [1]. With a such an asymmetry between potential cost and benefit, regulation can make the most difference to the long-term growth rate if the resource in question is only available a similarly small fraction of the time. More generally, and at least to a first approximation, it is obvious that gene regulation only is useful if the environmental conditions vary with time. Experimental data show that a repressive mode, where the presence of a resource disables the binding of a repressor to the DNA, is preferred when demand for expression is rare, whereas an activating mode is preferred in the opposite situation [8]. For the *lac* system and realistic time variations, the entropic effects appear to be so large that only a repressive regulatory mode is possible [9].

**Figure 1:**
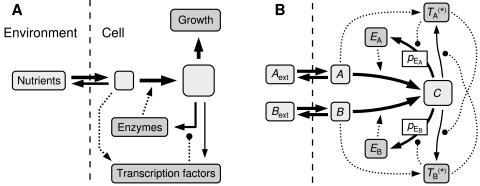
A) A general view of an organism with one or more metabolic pathways. Solid lines represent the flow of biomass, while dotted lines show regulatory interactions, with arrows for positive regulation and circles where the sign of the regulation can be varied. Nutrients are transported into the cell, processed by enzymes, and spent on cell growth and on replenishing the enzymes. Optionally, transcription factors may detect the presence of nutrients and regulate the enzyme production accordingly. B) A sketch of the most complete model considered here, where two nutrients, A and B, are turned into C by their respective enzymes, E*_X_*. Nutrients inside the cell activate the transcription factors, T*_X_*→T^*^ *_X_*, which in turn activate or repress the production of enzymes and each other. For parts of this paper, pieces of the model are removed, including the entire B side. When the transcription factors are excluded, the rate constants *p*_E_A__ and *p*_E_B__ control the production of enzymes.

To concretize the question of how to regulate metabolic processes, we consider the simplified view presented in figure 1(A). Here, an organism grows in a medium with one relevant rate-limiting resource, presumably a combination of carbon and energy, and several nutrients may be available to provide this resource. Nutrients present in the medium can be absorbed by the cell and converted into a useful form by the actions of different enzymes and other proteins. The proteins are replenished at the cost of slower growth, and evolution will optimize the growth rate over a set of environmental conditions by tuning the regulation of the protein production rates.

In this paper we study how the levels of one or two nutrients interact with the transcriptional regulation of their respective metabolic pathways to determine the growth rate of an organism under steady state conditions. Transient responses are an important aspect of metabolic regulation (see, e.g., [1], [3], [10]), but it is also hypothesized, and in some cases known, that microorganisms excel at optimizing their growth rate in the steady state [2], [11]. Because of this, we may approximate the fitness of an organism from its growth rate in a set of static environments. From such a treatment we can hope to gain further insights into how evolution chooses a mode of regulation, if any, depending on the environments that a species is exposed to.

## Results and Discussion

### The model

We have implemented a minimalist model of an organism that grows by metabolizing one or more compounds found in its environment. These compounds could represent different sugars, alcohols, or other nutrients, and although the model is not tied to any one particular example, we have glanced at the *lac* operon of Escherichia coli and the carbon metabolism of yeast. The model as such is rather similar both in spirit and form to that of Shoemaker et al. [12]. We have, as far as possible, derived equations and constants from first principles, in order to maximize the generality of the results. Some assumptions and approximations greatly simplify the model:

- Chemistry can be dealt with in terms of rate equations.
- Michaelis–Menten kinetics [13] are always appropriate.
- The cell contents are homogeneous, and transport processes are purely diffusional.
- Cell growth and division are one continuous process, resulting only in the dilution of the contents of the cell.
- Molecular concentrations have no hard upper limit.

Furthermore, when the environment is held constant, it is natural to seek steady state solutions to the equations. If only one stable solution exists, we use the corresponding growth rate as a fitness measure for the organism.

The differential equations that define the model can be found in Materials and Methods. Their parameters are largely given by basic physics and rudimentary knowledge about living cells, although in some cases only to within a few orders of magnitude. It should be pointed out that no attempt has been made to fit the model to experimental data, so although predictions made from the model may be qualitatively sound, they cannot be expected to agree quantitatively with any particular organism.

Figure 1(B) summarizes the interactions of the model, of which we will first consider a reduced version. A compound to be catabolized, call it A, exists outside the cell at concentration *A*_ext_, and can diffuse into and out of the cell. Inside the cell, A is converted into metabolite C with the help of an enzyme, E_A_. The metabolite C is what the cell needs in order to grow and make more E_A_. If A represents glucose, then C might represent pyruvate and ATP, and E_A_ is a whole set of enzymes, transporters, and other proteins. The generality of Michaelis–Menten kinetics as an approximation for one-way reactions (see, e.g., [14]) is what keeps this vagueness acceptable. In particular, note that even though we speak of E_A_ as an enzyme, the same rate equations arise if the main rate limiting step involves transport across the plasma membrane. Hence our minimalist model is not at odds with the more detailed model of Barford et al. for sugar uptake by yeast [15], given a sensible choice of parameters for the kinetics.

Let *A* denote the level of A, and so on. The production of C follows Michaelis–Menten kinetics, depending linearly on each of *E*_A_ and *A* but leveling off at high *A*. The rates of protein synthesis and cell growth are limited by C at low nutrient levels, but by translation and DNA replication, respectively, at higher C. Just how great the production of E_A_, *p*_E_A__, is depends on the rate constant *p*_E_A__, which captures both transcriptional regulation and the amount of protein synthesized per mRNA. Degradation of E_A_ is assumed to occur in the form of exponential decay with a half-life on the order of an hour.

### Optimal enzyme production rate

Even with a single nutrient, before attempting to regulate the enzyme production rate, we need to look at how much difference this can make to the growth rate over the whole range of environments. Only then can we know if and when there can be any point in employing transcriptional regulation.

**Figure 2:**
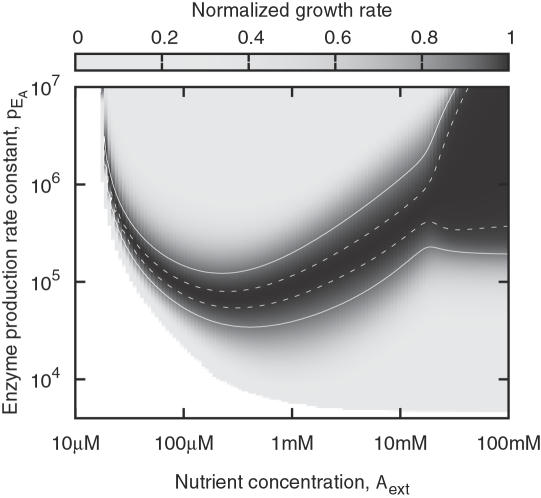
Growth rate as a function of the enzyme production rate constant, *p*_E_A__, over a range of nutrient concentrations. Dark areas mark where the growth rate is maximized for the respective *A*_ext_, and the solid and dashed white lines indicate 90% and 99% of the maximum, respectively. Values of 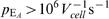 are not physically realizable, but are included to show the insensitivity to *p*_E_A__ at high *A*_ext_. Note the nonlinear brightness scale, which accentuates small deviations from the maximum.

All model parameters except the rate constant for enzyme production, *p*_E_A__, are fixed, and regulation amounts to adapting *p*_E_A__ to the nutrient concentration, *A*_ext_. Hence, we have explored how the steady state growth rate depends on *p*_E_A__ for many fixed values of *A*_ext_, by integrating the equations of the system from some initial state until reaching a fixed point. The growth rate thus found is normalized such that a value of 1 corresponds to the best possible growth rate for a given *A*_ext_. This quantity, which is shown in figure 2, is a fitness score that reflects how well an organism can compete with others that are perfectly adapted to a single environment. A tiny difference in growth rate becomes significant over many generations. Therefore, the gray scale in figure 2 was chosen to resolve small deviations from the maximum growth rate.

**Figure 3:**
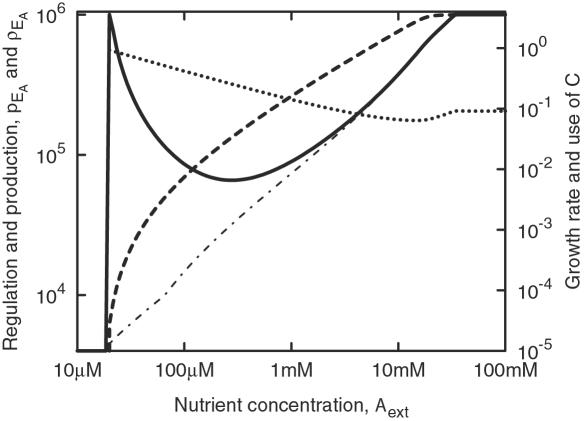
The transcriptional regulation, *p*_E_A__, that maximizes the growth rate of figure 2 (solid) and the corresponding actual enzyme production rate, *p*_E_A__ (dot-dashed), in units of molecules per cell and second. Also shown are the growth rate as fraction per hour (dashed, right scale) and the cost of producing the enzyme E_A_, expressed in terms of the total resource expenditure (dotted line, right scale).

Interpreting figure 2 is easier if we also consider figure 3, which shows the optimal *p*_E_A__ and the resulting protein production rate, *p*_E_A__ (determined by *p*_E_A__ and *C*), as functions of *A*_ext_, along with the growth rate and the fraction of resources (C) spent on enzyme production as opposed to growth. At very low concentrations, the cell barely has enough of the nutrient to survive, and it must focus almost all its energy on building enzyme. Because of the degradation of E_A_, there is a point (near *A*_ext_ = 20 µM) where each molecule of E_A_ costs more molecules of C to synthesize than it can bring about during its lifetime, and starvation is inevitable.

When the nutrient is more abundant, the resulting rise in *C* will lead to greater enzyme production. Our model assumes a linear relationship between *C* and the protein synthesis rate for low-to-moderate *C*, but the dash-dotted line in figure 3 indicates that with our model the enzyme production should approximately follow *C* ^0.6^ to maximize the growth rate. The exact relationship is hardly significant, but in any case it seems unlikely that the overall rate of protein synthesis would be adjusted to meet the needs of a single protein, and it is up to *p*_E_A__ to bridge this gap.

With higher enzyme concentrations it is primarily the diffusion of A into the cell that limits growth. There is a range of intermediate *A*_ext_ for which the optimal value of *p*_E_A__ varies relatively little, but with even higher *A*_ext_ and faster growth comes greater dilution of the enzyme, and this forces *p*_E_A__ to rise. If *A*_ext_ is higher still, the cell divides as fast as it possibly can and yet has much C to spare. As we do not take into account that protein synthesis may tie up machinery also needed for rapid growth and cell division, there is no incentive to keep *p*_E_A__ down in this limit. Thus, for very high *A*_ext_ the optimal value of *p*_E_A__ is the maximum allowed by the model. At the same time, the growth rate is insensitive to the value of *p*_E_A__; when resources are plentiful, how they are spent is less important.

We see that the fitness conferred by the metabolic pathway depends strongly on the enzyme production rate constant *p*_E_A__, except if the nutrient level is always kept within a limited range. Such static conditions may apply to some obligate symbionts, which can indeed lack the ability to regulate genes involved in important metabolic processes. For example, bacterial symbionts in aphids have lost most of their transcriptional regulation of amino acid synthesis [16], in a situation analogous to the one studied here in that the fitness gain from regulation must be weighed against the cost over a range of external conditions. For the vast majority of single-celled organisms, the environment can not be expected to be so constant. Selection pressure necessitates regulation of metabolic pathways.

### Transcriptional regulation

To incorporate transcriptional regulation into the model, we add a transcription factor, T_A_, which like E_A_ is produced from C. T_A_ can act as an enhancer or repressor for the production of E_A_, with the sign and strength of the regulation as two independent parameters (see Materials and Methods). However, T_A_ can only function when activated by the presence of A. The fraction in the active form is directly determined by the level of A as compared to a half-maximum parameter. Now *p*_E_A__ is no longer a constant, but a function of the levels of T_A_ and A. In addition to the dilution caused by growth, T_A_ is degraded, albeit at a much lower rate than E_A_. It would be quite natural to add autoregulation to T_A_, to allow it to stabilize its level over a wider range of growth rates or possibly be more sensitive to *A*_ext_, but doing so would make the results less transparent.

**Figure 4:**
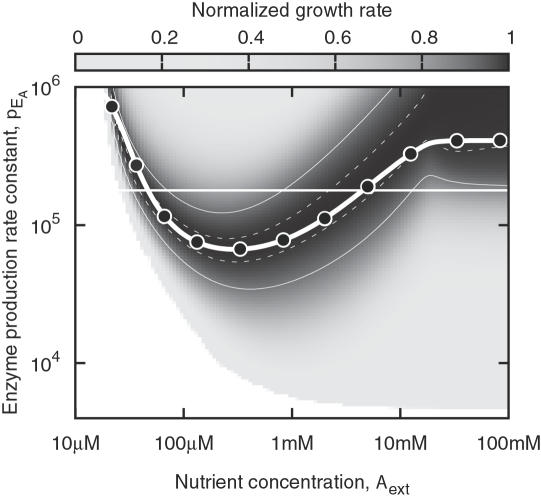
The behavior of a cell with well-tuned transcriptional regulation of the enzyme production. The normalized growth rate (see figure 2) was optimized for those *A*_ext_ indicated by points, with an equal number of generations spent in each environment. The resulting system responds to *A*_ext_ as indicated by the thick curve. For comparison, the straight white line shows the best *p*_E_A__ in absence of regulation.

By wiring and tuning a transcriptional network, evolution will tend to maximize the average growth rate over a succession of environmental conditions. In the limit where the environment very rarely changes, and the organism spends long time intervals in each setting, only the distribution of conditions matters, not the transitions between them. To keep things simple, we have defined the fitness of an organism as the mean of the normalized growth rate across an arbitrary selection of nutrient levels, as shown in figure 4. This fitness corresponds to the average growth rate when a similar number of generations is spent in each of these environments. In other words, the amount of time spent at each *A*_ext_ is inversely proportional to the dashed line in figure 3; not an entirely unreasonable first assumption.

In our equations, the transcriptional network is defined by five parameters, and taking the view that evolution will have had ample time to globally optimize a system with so few parameters, we have used simulated annealing to pinpoint the parameter values that maximize the fitness measure. The thick line in figure 4 shows the effect that transcriptional regulation has on *p*_E_A__, and thereby on enzyme production, for the network of optimal fitness. The points mark the *A*_ext_ through which the fitness measure is defined, and the color filling the points indicates that the achieved fitness is almost indistinguishable from the maximal one, for those *A*_ext_ that we have optimized for. A brief explanation of how the evolved transcriptional network operates is in order.

The transcription factor has become a repressor for the enzyme, and the downward slope for low-to-moderate *A*_ext_ is caused by the steadily more produced and activated T_A_ repressing the expression of E_A_. Some transcriptional leakage occurs, and there is never that much T_A_ present, so *p*_E_A__ is kept from falling too low. This explains how an increase in *A*_ext_ can result in a well-adjusted decrease in *p*_E_A__. Perhaps more surprising, then, is the increase that *p*_E_A__ shows for high *A*_ext_. The explanation: the time scale for decay and regeneration of T_A_ is roughly one day, and when the generation length is shorter than that, the level of T_A_ drops because of dilution.

In all, the transcriptional network is extremely good at maximizing the fitness. Without regulation, the best possible fitness is 0.81 (with *p*_E_A__≈1.8·10^5^), but with regulation the fitness rises above 0.99. Adding autoregulation to the transcription factor will further increase the fitness. We have also examined the effect of limiting the range of nutrient concentrations to between 100 µM and 1 mM. Then, the fitness scores of the best unregulated and regulated systems are 0.997 and 0.9996, respectively. Finally, forcing the regulatory mode to be activating was seen to substantially reduce the fitness score. Even with *A*_ext_ limited to values above 1 mM, the regulatory system was then incapable of giving a strong positive response to counter the dilution effect. We interpret this as a sign that the activating mode of regulation requires the transcription factor T_A_ to be positively autoregulating, or its activation by A to be nonlinear, or both. In either case, there are added requirements on T_A_, which may make activators more difficult to evolve. In addition, it could them more sensitive to random mutations, which would quantitatively affect the results of [9].

The difference in average growth rate due to regulation should be compared to the effect of adding the gene for the transcription factor to the genome of the organism, an effect not included in our model. Adding one gene should slow the growth rate by no more than one part in 1 000, and often far less (see Materials and Methods). This slowdown is far outweighed by the boost that regulation brings, as long as the nutrient level is not too constant. We conclude that when the nutrient level varies, transcriptional regulation of the metabolic pathway carries great benefits for the organism.

### An additional nutrient

Gene duplication plays a major role in the evolution of new functions. A duplicated enzyme or transcription factor will, if not lost, likely be subject to subfunctionalization [17], [18], a process that may in turn lead to neofunctionalization [19], [20]. To extend the model to the case where a second nutrient is present in the environment, we mimic a gene duplication event by adding a set of variables where “A” is replaced with “B”. Thus B is the alternative nutrient, turned into C by enzyme E_B_, whose production is governed by the rate constant *p*_E_B__. Clearly C should then be seen as the first common point along the metabolic pathways of A and B. Generally, A and B need not be equally useful for producing C, and the parameters that describe E_A_ and E_B_ may be quite different. Such a state of affairs may, e.g., apply to the preference for glucose over fructose in yeast. Still, we will assume that all the relevant parameters are equal between A and B, because a small difference between them should only distort our conclusions slightly, for instance by shifting the point of preference for one nutrient over the other.

With the introduction of the second nutrient comes an increase in the dimensionality of the state space that makes it impossible to visualize the full gamut of the system. However, we have observed that for any given environment the growth rate behaves nicely, with a single, rounded peak around some optimal *p*_E_A__ and *p*_E_B__ (data not shown). The optimum with respect to one parameter depends very weakly on the value of the other. It is therefore meaningful to keep *A*_ext_ and *p*_E_A__ fixed while studying the behavior as a function of *B*_ext_ and *p*_E_B__. Under the assumptions of our model, it is not necessarily optimal for the organism to metabolize the most abundant nutrient only, in contrast to the optimal strategy in [3]. The difference here is that when a nutrient is metabolized, its level is inside the cell drops due to the finite diffusion rate.

**Figure 5:**
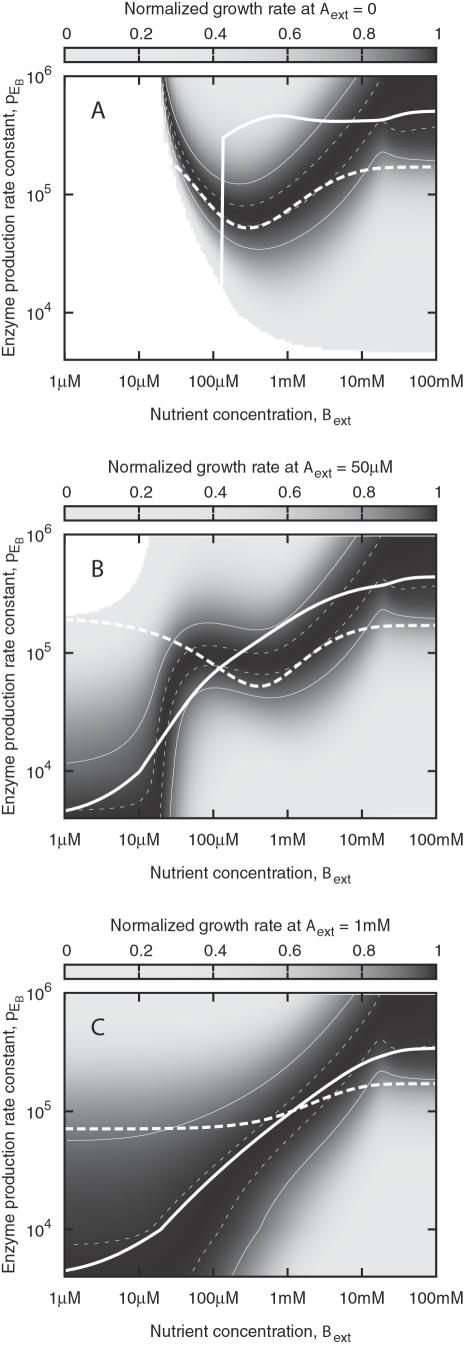
Growth rate as a function of the activation of a second metabolic pathway, presented as in figure 2. In each plot, the level of metabolite B is varied, while metabolite A is present at a constant level, and *p*_E_A__ is pegged at its optimal value in absence of B. The sharp transition of the optimal *p*_E_B__ in (B) signals a need for nonlinearity when *p*_E_B__ is actively regulated. The thick lines indicate what the simple transcriptional network of figure 1(B) can accomplish, when optimized over a wide range of environments, either emphasizing high (solid) or low (dashed) nutrient levels. Comparison with figure 4 shows that it is far more difficult to achieve near-optimal regulation when the second metabolic pathway is added.

Figure 5 shows how the growth rate depends on *p*_E_B__ for different *B*_ext_, with *A*_ext_ fixed at 0, 50 µM, or 1 mM and *p*_E_A__ optimized at *p*_E_B__ = 0 (to *p*_E_A__ = 0, *p*_E_A__≈1.5·10^5^, or *p*_E_A__≈9·10^4^, respectively, as per figure 3). As expected, the figure reveals that when little B is available, it is best for the cell not to bother with B at all. However, when *B*_ext_ comes within a factor of about ten of *A*_ext_, the optimal *p*_E_B__ rises quickly, from zero to a value near where the optimum would be in the absence of A. That is, when *B*_ext_≳*A*_ext_, the dependence of *p*_E_B__ on *B*_ext_ is described by figure 5(A) (which is identical to figure 2). For other values of *A*_ext_ than those shown in figure 5, the only notable trend is that the transition region is narrower and closer to *A*_ext_ at low *A*_ext_. An organism that is well adapted to a slowly changing environment will show a strong, often nonlinear response to the level of B in the region where 3≲*A*_ext_/*B*_ext_≲30.

### Bistability

After a change in the environmental conditions, and after transients have died down, the internal state of a cell may depend on the history of the cell, rather than on the new environmental conditions alone. Bistability, the coexistence of two stable fixed points in the dynamics, implies such hysteresis, because the initial state of the system generally determines which fixed point it goes to. In principle, stochastic effects always make the two states metastable, but the time scale for spontaneous switching may vary greatly. See [21] for a review.

Evolution does not indiscriminately optimize the steady state growth rate. The environment changes from time to time, and if moving to a different operating region transiently carries a cost in the form of slowed growth, that cost must eventually be recuperated. Depending on the typical pattern of changes in the environment, the best solution for the individual cell may involve hysteresis. In clonal populations, phenotypic heterogeneity caused by bistability can be advantageous to the genome if it allows at least part of the population to survive a disaster [22]. Genes can thus hedge their bets by making individuals take risks, but only if a timely response is impossible is this a better strategy than for the cells to individually adapt to new conditions [23].

The *lac* operon of *E. coli* is a well-known example of a regulatory module tasked with deciding whether to make use of a specific metabolite. This system, in short, contains a positive feedback loop that activates lactose transport and metabolism when a metabolite of lactose is detected, but only if the glucose level in the environment is comparatively low. Experiments have shown that the *lac* system has the potential for bistable behavior when subjected to artificial inducers [24]. This property of the system is appealing, and we can picture how it could fit into our results in figure 5. Although the optimal *p*_E_B__ does not follow an S-shaped curve, the steepness and width of the region where *p*_E_B__ switches are, at low *A*_ext_, great enough to allow for bistability at a relatively low fitness cost in the steady state.

However, it appears that the response of the *lac* operon to lactose itself, as opposed to an artificial inducer, is only a steeply graded monostable function, not a bistable one. A convincing explanation is that with time-varying lactose levels, a graded response provides significantly faster switching between operating modes [10]. Based on figure 5, we posit that a graded response is advantageous also when the lactose level varies very slowly, because unless the *lac* system is dissimilar to our model in some unforeseen way, the optimal *lac* expression is ever a smooth function. Consequently, we expect bistability in this context to be a rare phenomenon.

### Transcriptional regulation revisited

Regulation of the production of the two enzymes, E_A_ and E_B_, requires signaling from both A and B, and we have seen that some degree of mutual exclusivity is desirable. If a new transcription factor, T_B_, were to be created by duplication of an autoregulating T_A_, it might be prudent to retain a full set of regulations, with T_A_ and T_B_ regulating each other, themselves, and both enzymes. However, this would introduce an excessive number of parameters and obscure the issue of how easy it is to achieve beneficial transcriptional regulation. Therefore, we again use a stripped-down network, this time one that only includes regulation of E_A_ by T_A_, of E_B_ by T_B_, and mutually between T_A_ and T_B_.

In the same spirit as for the one-nutrient system, we have optimized the fitness of the regulatory network over two different sets of *A*_ext_ and *B*_ext_, differing in their emphasis on high-nutrient or low-nutrient environments. The thick lines in figure 5 illustrate how the resulting networks perform, which in either case certainly is better than with any constant value for *p*_E_B__.

When emphasis is placed on high-nutrient emphasis, the two transcription factors become mutual repressors, as one may have expected. This makes bistability possible even without autoregulation, but only when we add autoregulation to the model or modify the parameters do we see bistable behavior (data not shown). The way we assess the growth rate in each environment implicitly assumes monostability by denying the system a history (see Materials and Methods), which is an extreme case of the idea outlined earlier: changes to the environment are infinitely rare, and bistability can evolve only because it never gets a chance to do any harm. Nevertheless, this emergence of bistability demonstrates a point: nontrivial behavior may appear even when not selected for, because of the constraints that evolution has to work with.

## Discussion

We have developed a model for how the steady state growth rate depends on the activity of metabolic pathways, including their transcriptional control, in an idealized organism. This represents an implicit way to model the evolution of transcriptional networks subject to simple metabolic tasks.

A key finding is that even when no alternative pathways exist, transcriptional regulation confers a substantial fitness advantage in all but the most static environments. In other words, transcriptional control is required for an organism to be competitive, even for a very simple metabolism. Furthermore, we have shown that this fitness advantage can be well exploited by a remarkably crude regulatory system, which relies on transcriptional repression. The observation that the mode of regulation correlates with the demand for expression for many metabolic genes [8] is explained by Savageau from the perspective of resilience to mutations [9], but we believe that our results point to a complementary explanation, namely that negative regulation is easier to accomplish. For genes that are rarely used, it is relatively important to minimize the resources spent on the regulatory system, as opposed to fine-tuning the expression level and dynamical behavior, and this would tend to favor repression over activation.

When two pathways are available, as would be the case when two catabolizable sugars are present, the highest growth rate is achieved by sharply activating the pathway of one nutrient, when the level of that nutrient nears the level of the other. At low nutrient concentrations this optimal response is distinctly nonlinear.

In line with previous work [10], we have demonstrated that bistability is not a desirable feature of metabolic pathway regulation, at least not in a steady state limit. Nevertheless, optimizing the fitness of a regulatory system can cause bistability at some nutrient levels, suggesting that such complex behavior can emerge even when it in itself is unfavorable, merely because the underlying mutations have a positive net effect on fitness. This is reminiscent of, yet different from, how pathway complexity can be increased by the actions of the evolutionary mechanisms per se [25].

We can also picture conditions that would promote bistable switching of metabolic pathways. From figure 5, one can imagine the existence of two distinct overall environmental states, whose respective ranges for the nutrient concentration overlap. If transitions between the two states are rare, and intra-state fluctuations of the nutrient level are too rapid for regulation to follow suit, bistability in the overlapping range can be expected to increase fitness, as it prevents needless state changes. Such a scheme could perhaps be applied to the lactose level in a chemostat with *E. coli* over many generations. As demonstrated in [1], the expression level of the *lac* genes can be significantly altered by fitness-raising mutations in a few hundred generations of growth in a constant lactose/glycerol medium. Thus it may well be possible to achieve bistability in the lactose response of *E. coli* through evolution in a laboratory setting.

Our model predicts that cells growing in a very low nutrient medium will have their nutrient uptake and catabolism up-regulated as compared to when the nutrient is more abundant. Such behavior has indeed been seen in yeast under glucose limited conditions [26], where the hexose transporter HXT6 and the glycolysis enzymes HXK1 and GLK1, along with several other glycolysis proteins, were found to be significantly up-regulated. Our results predict that this effect only comes into play at sustained growth close to the long-term starvation limit, and only when there is no appreciable level of alternative substrates present.

Our predictions are primarily qualitative, not quantitative. For example, the exact location of the point where the model breaks down in figure 2 should not be considered as realistic, as the degradation rate of the enzyme is higher than it would be in an organism adapted to very low nutrient levels. Still, a real microorganism may survive periods of starvation through sporulation and other mechanisms outside our model, but will eventually die if the nutrient levels are too low.

## Material and Methods

We here give the equations and parameters of the full model with two nutrients and transcriptional regulation. For the smaller models discussed in the text, parameters are set to zero as appropriate. C is the universal currency of our cell, and takes on the roles of energy carrier and building block. Being small molecules, A, B, and C have a mass of about *m*_A_ = 100 Da. The proteins E and T weigh in at 10^3^ *m*_A_, and synthesis of one E or T consumes *s_protein_* = 10^4^ molecules of C. Let *C*, *A*, *E*_A_, *T*_A_, etc. denote the number of molecules in a cell of fixed volume *V_cell_* = 1 µm^3^. Brownian motion sets a limit of about one collision per molecule pair per second, and this affects many parameters. For brevity we let *X* represent A or B where their equations are identical.

Transcription factor activation:
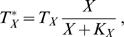
where *K_X_*∈[10^2^,10^6^]*V* ^−1^ *_cell_* (or 1.7·10^−7^,10^−3^M), so activation can be made very sensitive to A or B.

Transcriptional regulation of enzyme production:
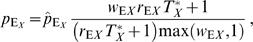
where the maximum rates 
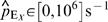
 are limited by ribosome count and speed. The parameters 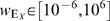 determine the type of regulation, ranging from strong repression to linear activation via leakiness and indifference. The strength of *T* ^*^ *_X_* as a regulator is 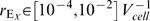, meaning that hundreds to thousands of TF molecules are needed for regulation. Similarly for the regulation of *p*_T_A__ by *p*_T_B__ and vice versa, *mutatis mutandis*.

Enzyme activity:
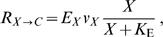
where *v_X_* = 10^3^ s^−1^ and *K*_E_ = 10^8^ *V* ^−1^ *_cell_*≈1.7mM, which loosely means that one in 1 000 collisions between *X* and E*_X_* leads to a reaction that takes 1ns.

Protein production, cell growth, and dilution:
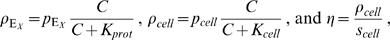
where *K_cell_* = 10^8^ *V* ^−1^ *_cell_* and *K_prot_* = 10^6^ *V* ^−1^ *_cell_* reflect bottlenecks in cell replication and protein synthesis, s*_cell_* = 10^10^ *V* ^−1^ *_cell_* because a cell takes that many molecules of C to build, and *p_cell_* = 10^7^ *V* ^−1^ *_cell_s* ^−1^ should give a maximum growth rate of about 20 minutes per generation.

The rate equations:
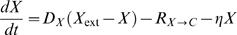


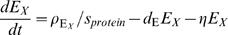


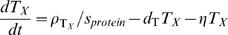


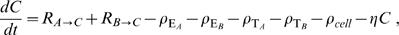
where *D_X_* = 1 s^−1^ lies between an upper limit set by diffusion and a lower limit set by the maximum growth rate, *d*_E_ = 10^−3^ s^−1^ makes E*_X_* rather short-lived but keeps its level reasonable even at the maximum production rate, and *d*_T_ = 10^−5^ s^−1^ is a realistic decay rate far slower than *d*_E_.

For an upper bound to the reproductive cost of adding an extra gene, consider a prokaryote for which the DNA replication rate is limiting. If this rate is proportional to the genome size, and there are 1 000 genes, the fitness cost is on the order of 10^−3^. By comparison, the cost of synthesizing the extra DNA is closer to 10^−5^ *s_cell_* .

To find the growth rate in the equations' steady state, we performed a numeric integration from the state with all variables zero except *C* = 10^8^ *V* ^−1^ *_cell_*, using the bsimp stepper from GSL (http://www.gnu.org/software/gsl/). For figure 5 we probed for bistability by gradually going from low to high *B*_ext_, and vice versa.
